# Novel concepts of cell-of origin in neuroendocrine neoplasms

**DOI:** 10.1007/s00428-025-04311-2

**Published:** 2025-11-14

**Authors:** Ilaria Marinoni, Simona Avanthay, Nicolas Alcala

**Affiliations:** 1 Institute of Tissue Medicine and Pathology, University of Bern, Bern, Switzerland; 2 Graduate School for Cellular and Biomedical Sciences, University of Bern, Bern, Switzerland; 3 Computational Cancer Genomics Team, Genomic Epidemiology Branch, International Agency for Research on Cancer, Lyon, France

**Keywords:** Neuroendocrine neoplasms, Carcinoma, Gastro-entero-pancreatic endocrine neoplasm, Lung neuroendocrine cancer, Cell of origin

## Abstract

Neuroendocrine neoplasms (NENs) are a heterogeneous group of tumors. The rarity of the disease, together with the lack of mutations in the classical tumor suppressor genes and the paucity of models, has impaired our understanding of the mechanisms of progression and the cell of origin of these tumors. Due to their higher frequency, this review focuses on Gastro-Entero-Pancreatic (GEP) and Lung NENs. While recent molecular profiling has shed light on the possible cell of origin of GEP- and lung NENs, many questions remain unanswered and further studies using proper *in vitro* and *in vivo* models are needed, combined with the latest technologies such as single-cell and spatial sequencing and deep-learning for digital pathology. Genomic and epigenomic evidence suggests that pancreatic NENs originate from adult pancreatic cells rather than common progenitor cells; however, ultimate proof *in vitro* or in *vivo* is still lacking. Similarly, emerging molecular evidence suggests that lung NENs may have very diverse origins, encompassing most lung cell types, but much work is still needed to pinpoint their cell of origin. Further, tumors with mixed endocrine and non-endocrine composition suggest the possibility of trans-differentiation and acquisition of neuroendocrine features in different cell types. This review aims to summarize emerging insights on this topic, highlight future directions for identifying the cell of origin of NENs in these organs and explore how this knowledge may ultimately translate into clinical advances.

## Introduction

Neuroendocrine neoplasms (NENs) are a heterogeneous group of tumors with widely varying morphologies and behaviors. NENs can be functional, characterized by overproduction of specific hormones which can cause severe syndromes, or non-functional with no clinical symptoms caused by hormone production. NEN incidence has been increasing in the last years due to better diagnostic tools and increased lifespan, and it is currently estimated as 7 cases out of 100,000 [1, 2].They are most frequent in the respiratory and gastro-entero-pancreatic (GEP) tract, but can occur in all organs, in particular the thymus, pituitary, adrenal gland, kidney, bladder, reproductive organs, skin, and breast [1–3].

The technological breakthroughs of the past decades have much improved our understanding of NEN carcinogenesis, nevertheless the lack of mutations in the classical tumor suppressor genes or oncogenes with consequent pathways dysregulation, together with the rarity of the disease and the limited number of *in vitro* and *in vivo* models impaired the understanding of mechanisms of initiation of these tumors.

Importantly, the identification of their cell of origin would be crucial for better diagnosis and therapy selection. Cell of origin identification provides important information about mechanisms and regulation of progression occurring in that specific cell type, with clear therapy implications. For example, in B-cell lymphoma the cell of origin classification helps tailoring treatment strategy and response prediction [4]. Yet, such an approach is not implemented in solid tumors and specifically in neuroendocrine tumors (NETs). However, recent data suggest that the cell of origin stratifies pancreatic neuroendocrine tumors (PanNETs) with different clinical characteristics, suggesting that such classification may be relevant also in NETs [5, 6].

Molecular analysis is increasingly used to identify the cell of origin and understand its role in tumor development and treatment. Advances in single-cell and spatial omics provide high-resolution data on cellular composition, evolutionary dynamics, and clonal relationships in tumors, which helps in identifying the cell of origin [7, 8]. However, the main limitations of the molecular analysis performed on tumor tissue are that they are often obtained at a late stage of the disease and fail to recapitulate the early steps of cancer development. Ultimate proof of the cells of origin in NET can come only from *in vitro* or *in vivo* models, while studies on tumor tissues are rather providing hypothesis on cell of origin.

This review outlines emerging insights into the cell of origin of NENs and their potential clinical implications. Given that NENs from different anatomical sites display distinct histopathological and molecular features, we structure the discussion by site, focusing on thoracic and gastro-entero-pancreatic (GEP) NENs, which are the most frequent and have seen the greatest recent advances in molecular classification and cell-of-origin studies. For each site, we discuss current histopathological and molecular subtypes, examine evidence on their cell of origin, and consider trans-differentiation between NEN subtypes. We conclude with future directions for uncovering the origins of NENs and translating this knowledge into clinical advances.

## Thoracic NEN

### Classification and molecular profiles

Lung NENs (also called pulmonary carcinoids) are currently classified into well-differentiated lung NET, G1 and G2 based on the proliferation activity, as measured by the number of mitoses and the presence of necrosis, and poorly differentiated NEC, itself subdivided into large cell and small cell carcinomas [9, 10]. Note that contrary to GEP-NETs, NET G3 would be very rare and are not recognized in the current classification, despite increasing evidence of their existence and clinical importance [11–14]. Lung NETs and NECs have been considered as two distinct diseases with different etiologies—NECs are strongly associated with smoking while NETs are not—and clinical behavior [15, 16]. Therapeutic strategies for lung NENs vary between NETs and NECs [17]. NETs treatment usually involves surgery and long follow-up to monitor recurrence, and occasionally additional treatments in case of metastatic spread. Small cell lung carcinomas are mainly treated with chemotherapy, while large cell carcinomas are treated by surgery and chemotherapy (and possibly radiotherapy) at an early stage, and by chemotherapy at an advanced stage.

Lung NECs and NETs also have distinct genomic drivers. Lung NECs are mostly universally driven by *TP53* and *RB1* alterations [16, 18–20], while lung NETs can have a plethora of drivers: *MEN1*, *EIF1AX*, and *ARID1A* losses, and a few cases with *BRAF* mutations have also recently been reported [21–23]. Recurrent chromosomal alterations such as chromosome 3 and 11 losses, and chromosome 5, 7, and 8 gains have also been reported. Interestingly, it has been shown that genomic alterations are strongly associated with molecular groups with different clinical characteristics, named Carcinoid (Ca) A1, A2, B, and supra-carcinoids. Tumors from Ca A1 have the best prognosis and are associated with chromosome 3 losses and *EIF1AX* mutations; Ca A2 tumors also have a good prognosis and are associated with chromosome 5 and 7 gains and *ARID1A* mutations; Ca B tumors have worse prognosis and are associated with *MEN1* mutations and chromosome 11 deletion in the *MEN1* region, and chromosome 5 and 7 gains [22]. Finally, supra-carcinoids have the worst prognosis and have transcriptomic and methylation profiles similar to that of high grade large-cell neuroendocrine carcinomas and encompass the few *BRAF* mutations reported in lung NETs [21, 23].

### Cell of origin of lung NENs

#### Lung NETs

Lung NETs are subdivided into molecular groups with strikingly different genomic, transcriptomic, and epigenetic profiles [22, 24], and interestingly, these molecular groups have also recently been shown to originate from different parts of the lung. Group Ca A1 tumors predominantly originate from the peripheral (distal) locations whereas Ca A2 tumors predominantly originate from endobronchial (proximal)/central locations [25]. Recent single-cell sequencing studies revealed a strikingly different cellular compositions along the proximal-to-distal axis of the respiratory system, with proximal locations mostly consisting of basal (median of 80%) and neuroendocrine cells (median of 15%), while distal locations had high levels of immune cells (e.g., medians of 7% of macrophages and T cells) and had more club, alveolar type I (AT1) and II (AT2) cells and less than 1% of basal and neuroendocrine cells [26]. The distinct locations of Ca A1 and A2 tumors may reflect differences in their cells of origin as well as in their etiologies, influenced by varying environmental (e.g., smoking, air pollution) and lifestyle risk factors across sites.

**Fig. 1:**
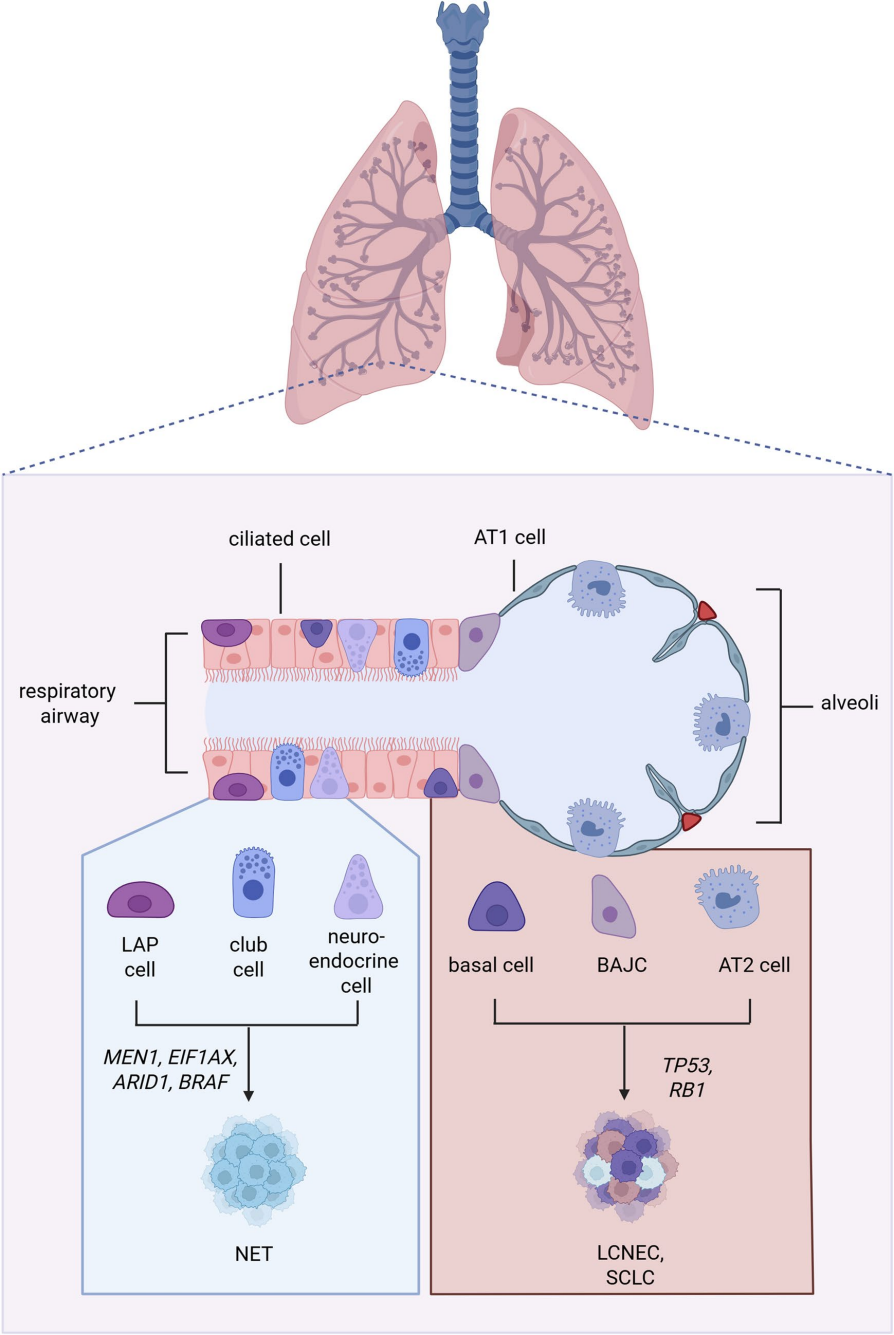
Possible cell of origin of lung NENs. Created in BioRender. (2025) https://BioRender.com/minklcr

Lung NET molecular groups were also shown to express variable levels of transcription factors and neuroendocrine markers, suggesting distinct neuroendocrine or non-neuroendocrine cells of origin (Fig. 1). For instance, group Ca A1 tumors expressed an important canonical neuroendocrine marker (ASCL1), absent from groups Ca A2 and Ca B. Ascl1 is a well-known master regulator of neuronal and neuroendocrine cell commitment and differentiation in vertebrates suggesting that Ca A1 tumors may originate from neuroendocrine cells [27]. Ca A2 tumors expressed transcription factor *HNF4A*, which is involved in neuroendocrine lineage specification in GEP cells [28]. The proximity of Ca A1 and Ca A2 with neuroendocrine cell states is further supported by deconvolution of single-cell neuroendocrine cell profiles, which showed that Ca A1 and Ca A2 tumors most resembled terminally differentiated neuroendocrine cells in terms of their gene expression profiles. In contrast, tumors from the Ca B group most resembled club cells, canonical epithelial airway cells that share a common progenitor with pulmonary neuroendocrine cells (PNEC) but undergo a secretory cell type differentiation [29]. Tumors from the supra-carcinoid group most resembled the undifferentiated lower airway progenitor cells [21], which are known progenitors of PNECs [30]. This suggests that NETs from this aggressive group might either originate from a different, less differentiated cell than other NETs, or that these tumors have the same cell of origin but undergo a dedifferentiation process. Methylation profiles further supported the fact that the different molecular groups have different cells of origin, as the groups were well-separated by methylation data alone [22].

Finally, almost all NET patients with diffuse idiopathic pulmonary neuroendocrine cell hyperplasia (DIPNECH) have been shown to have Ca A1 tumors [25]. This suggests DIPNECH as a potential precursor lesion of Ca A1 tumors, and that this group might have a diseased cell of origin with a specific phenotype and altered cell state.

Because of the paucity of in vitro and in vivo models for lung NETs and PNECs, limited biological understanding of neuroendocrine cells, and the recency of molecular profiling studies, experimental evidence on the cells of origin of lung NETs is still lacking. Importantly, PNECs are notoriously difficult to culture, due to their rarity, the absence of isolation protocols, and difficulties with sustaining long-term cultivation. However, novel *in vitro* models are emerging that could empower such studies in the future [31].

In summary, the literature suggests various possible cells of origin of lung NETs. More studies, in particular using single cell sequencing of NETs, normal neuroendocrine cells, and diseased tissues (DIPNECH), as well as experiments aiming to reproduce NET formation *in vitro*, will be needed to elucidate the mystery of their cell(s) of origin [31].

#### Lung NECs

Lung carcinomas- large cell neuroendocrine carcinoma (LCNEC) and small-cell lung carcinoma (SCLC)- are believed to originate from certain respiratory epithelial cells. Indeed, experimental data from genetically modified mouse models of SCLC where co-deletion of *TP53* and *RB1* was performed suggest that multiple cell types can give rise to SCLC, including neuroendocrine precursor cells, AT2 cells, basal cells, and bronchioalveolar junction cells **(**Figure 1) [32, 33]. Importantly, different cells of origin in interaction with various driver events might be the underlying cause of distinct carcinoma subtypes and interpatient heterogeneity [17, 33, 34]. It has been proposed that the loss of Rb1, which controls neuroendocrine differentiation, could influence the transition of epithelial cells such as progenitor-like AT2 cells to a neuroendocrine -like cell state [35]. A recent preprint presenting results from genetically engineered animal models of SCLC shows that tumors originating in basal cells, but not in neuroendocrine cells, recapitulate human SCLC. These results further support the possibility that SCLC may primarily originate from basal cells [36].

Much less is known about the cell of origin of LCNEC, and evidence comes from tumor molecular analyses rather than direct *in vivo* or *in vitro* experimental data. Given the shared molecular characteristics of LCNEC and SCLCs, in particular genomic drivers *TP53* and *RB1* that seem involved in the acquisition of neuroendocrine cell state in SCLC as outlined above, it is speculated that LCNEC and SCLC have similar cells of origin (Figure 1) [17]. This is further supported by the similarities in gene expression programs between SCLC and LCNEC, with the discovery of “SCLC-like” LCNEC that cluster with SCLC based on their expression profile, and “LCNEC-like” SCLC that cluster with LCNEC (RB1-altered LCNEC) based on their expression profile [19]. Nevertheless, the transcriptomic differences among LCNEC warrant further studies to verify whether they may be due to different cells of origin. Indeed, while the LCNEC type I molecular group presents a neuroendocrine profile including high expression of *ASCL1,* the LCNEC type II group presents reduced expression of neuroendocrine markers [19]. Whether this is a result of cells of origin with different levels of neuroendocrine differentiation or the result of a dedifferentiation process after LCNEC initiation is currently unknown.

Overall, these results suggest that cell plasticity, in particular with regards to neuroendocrine cell (de-)differentiation, might be pervasive and the cause of the difficulties in pinpointing the exact cell of origin of the different NEC entities. More work, including with more realistic experimental models and high-resolution sequencing technologies, in particular for LCNEC, will be needed to decipher cell-state trajectories throughout the course of the disease.

#### Trans-differentiation in lung NENs

In the lung, trans-differentiation of carcinomas (adenocarcinoma and squamous cell carcinoma) toward SCLC following treatment is the most frequently reported trans-differentiation event [17]. This occurs in particular in EGFR-mutated non-small-cell lung cancer (NSCLC) when resistance to anti EGFR treatment develops [37]. This shows that neuroendocrine differentiation is plastic and can be acquired by cells from different origins, including cells potentially derived from alveolar cells. Trans-differentiation between LCNEC and SCLC is also suggested by the existence of combined LCNEC and SCLC tumors, mostly involving Rb1 deficient tumors where the absence of Rb1 was shared between the LCNEC and SCLC compartments, suggesting a common origin [19]. Between NETs and NECs, a recent study suggests that in never smokers, SCLC could originate from a trans-differentiation from lower grade NETs through chromothripsis—a chromosomal aberration event where several chromosomes are shattered in a single-cell division and reassembled by the DNA repair machinery—forming extra-chromosomal DNA where oncogenes *CCND1* or *CCN2/CDK/MDM2* are strongly amplified [38]. This is in line with work that demonstrated the existence of entities crossing the barrier between Lung NET and -NEC, such as supra-carcinoid with the morphology of Lung NETs but the molecular and clinical characteristics of Lung NENs, in particular LCNEC [22].

## GEP-NEN

### Classification and molecular profiles

GEP-NENs are classified into well-differentiated NETs, grade G1, G2 and G3 based on the proliferation index, and poorly differentiated neuroendocrine Carcinoma (NEC) with a more aggressive behaviour [39, 40]. Therapeutic strategies for GEP-NENs depend on their grade and size. Usually it includes surgery and long-acting somatostatin analogues, everolimus, sunitinib, or chemotherapy for NET while the first-line treatment for NECs is instead platinum-based chemotherapy [41]. GEP-NET and NEC have distinct genetic drivers. For example, in the pancreas, NETs have recurrent mutations in *MEN1 (*MENIN), *DAXX (*Death domain associated protein*) , ATRX* (Alpha Thalassemia X-linked syndrome) and in genes encoding for mTOR pathways, while NECs often show *TP53* mutations and RB1 loss [42] However, while the genetic background of pancreatic NENs is well characterized, genetic drivers of other GEP-NET are less understood and often unknown. In Small Intestinal NETs (Si-NETs), only 10% of patients carry mutations in the gene *CDKN1B* (*p27*). Other rare mutations are found in *BCOR, FAT1, MUC5AC,* and *MCAM* [43]. PanNETs also present recurrent Copy Number Variants (CNVs). Indeed, based on CNVs, PanNETs can be divided into three groups [44, 45]. One group is enriched in PanNETs with recurrent loss in 10 chromosomes (11, 21, 10, 16, 2, 22, 6, 1, 8 and 3) and mutations in *MEN1*, *DAXX* and *ATRX.* A second group displays fewer CNVs except for loss of chromosome 11 and *MEN1* loss of heterozygosity. PanNETs in this group are mainly mutated in *MEN1*. A third group presents variable aneuploidy with different gain and loss [44].

### Cell of origin of GEP-NEN

#### Low grade PanNENs

Low grade PanNENs encompass G1 and G2 PanNETs. Even though their cell of origin is still not fully elucidated, both experimental evidence and tumor molecular analyses suggest they arise from adult pancreatic islets cells, which include 5 different types of cells: α-, β-, γ- (or PP), δ - and ε-cells, producing respectively glucagon, insulin, pancreatic polypeptide, somatostatin and ghrelin, rather than from progenitor or stem cells [5, 46].

Studying the early steps of tumor development allows us to gain insights into cell of origin. While precursor lesions are difficult to diagnose in sporadic tumors, patients affected by familial MEN1 syndrome present multiple lesions in the pancreas including microadenomas (microtumors) which can provide important information about the tumor initiation process. Hormone expression of these microscopic tumors showed that 53% express glucagon (produced by α-cells), 20% Pancreas Polypeptide (produced by γ-cells) and 15% express insulin (produced in β-cells) [47]. This distribution does not represent the abundance of respective islet cells, suggesting an increased susceptibility of α- and γ-cells towards *MEN1* mutations. Interestingly, recently a common progenitor cell was proposed for α- and γ-cells based on single cell data [48]. The susceptibility of α-cells to *MEN1* mutations is also confirmed by the fact that sporadic insulinoma originating from β-cells rarely show mutations in *MEN1*, rather they are mutated in the transcription factor *YY1* in 25% of the cases [49]. Non-functional PanNETs (NF-PanNETs) instead present mutation in *DAXX* or *ATRX* in almost 40% of cases, often in association with *MEN1* [50]. Insulinomas express PDX1, a transcription factor involved in β-cell differentiation, while most NF-PanNETs are ARX-positive which is instead regulating α-cells differentiation [5, 51, 52]. These data suggest that NF-PanNETs originate mainly from α-cells while insulinomas originate from β-cells. Interestingly, DAXX/ATRX loss is observed only in malignant metastatic insulinomas which also express ARX, inconsistent with normal β-cell differentiation [51], and indicating instead a possible trans-differentiating from α- to β-cells. Similarly, glucagonomas developing in α-specific *Men1* knock-out trans-differentiate to insulinomas in advanced stages of the disease [53]. Intriguingly, transgenic *Men1* knock-out mice mainly develop insulinoma regardless of whether the knock-out occurs in progenitor cells of both endocrine and exocrine pancreas (*Pdx1*), in β-cells or in α-cells, supporting the hypothesis of multiple cells of origin [53–55]. These data underline possible differences between Menin role in mice and humans.

**Fig. 2:**
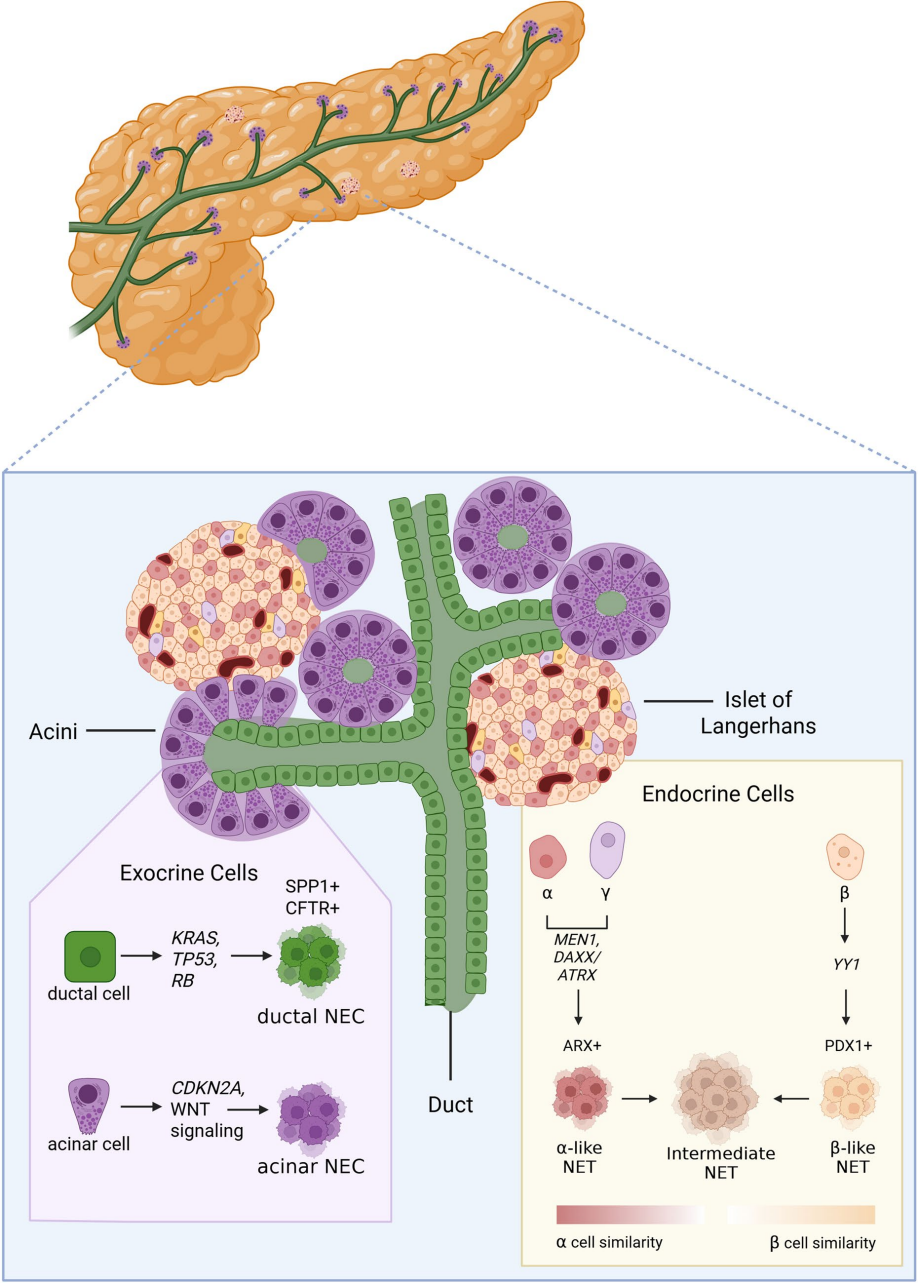
Possible cell of origin of pancreatic NENs. Created in BioRender. (2025) https://BioRender.com/minklcr

Epigenetic profiles are shaped during cell differentiation, and they can be used to identify cells of origin across different tissues or cancer types [56]. Recently, H3K27ac super enhancer profiles identified two major subtypes of PanNETs [52]. These include α-like tumors that express ARX and β-like tumors that express PDX1. Rarely, PanNETs show expression of both transcription factors or neither. Similarly, DNA methylation stratifies PanNETs into three groups: β-like tumors, epigenetically similar to β-cells, α-like tumors epigenetically close to α-cells, and intermediate tumors which progressively loose specific cell differentiation yet maintaining α-like features (Figure 2) [5]. β-like PanNETs are mainly insulinomas, clearly originating from β-cells, with distinct β-cell transcription factor expression. α-like and intermediate tumors express ARX, suggesting that they may originate from α-cells. α-like tumors are enriched in *MEN1* mutations, they have low copy number variation, and they are usually small with good prognosis while intermediate tumors are enriched for *MEN1* and *DAXX/ATRX* mutations (ADM), they have high copy number variation, they are large and with high risk of relapse [5]. These data suggest progression from α-like tumors into intermediate ADM upon acquisition of *DAXX/ATRX* mutations. Intermediate tumors occasionally express stem cell markers, however, stem cell origin remains unproven. Transcriptomic analysis revealed a group of PanNET with an aggressive phenotype and progenitor-cell-like signature, Metastasis Like Primary Tumors (MLP1), and progressive loss of β-cell differentiation [57, 58].

#### High grade PanNENs

High grade PanNENs include well-differentiated G3 PanNETs and poorly differentiated pancreatic neuroendocrine carcinomas (PanNECs). Well-differentiated G3 PanNETs exhibit genomic drivers similar to low grade PanNETs including *MEN1*, *DAXX*, *ATRX*, *PTEN*, *TSC2,* as well as specific ones such as *TP53*. PanNECs instead present mutations similar to pancreatic ductal adenocarcinoma such as *TP53*, *KRAS*, *BRAF*, *PIK3CA*/*PTEN* [59], suggesting a possible distinct cell of origin compared to PanNET, followed by trans-differentiation toward a neuroendocrine phenotype. DNA methylation profiles efficiently discriminate PanNECs from PanNETs G3. Indeed, while G3 PanNET are epigenetically similar to α-cells, PanNECs are closer to acinar cells, underlying a possible different cell of origin. Consistently, PanNEC showed positivity for SOX9 and loss of expression of the endocrine transcription factors ARX and PDX1. SOX9 has an important role during pancreas development. During differentiation SOX9 is expressed in multipotent progenitor cells while in adult pancreas only ductal and centro-acinar cells maintain the expression [60, 61]. A subset of PanNETs also expresses SOX9, suggesting that also NET may lose endocrine differentiation during the progression while acquiring progenitor-like features. Lately it has been suggested based on mutational and epigenetic profiles that PanNECs consist of two distinct groups: those originating from acinar cells and those originating from ductal cells (Figure 2) [62]. Ductal PanNECs express markers of the duct lineage such as SPP1 and CFTR and they carry mutations in *KRAS* and *TP53* and *RB* loss. The expression of the transcription factors SOX2 and ASCL1 together with increased chromatin accessibility at the corresponding locus also characterizes these subtypes [62]. SOX2 is expressed during the development in embryonic stem cells and neuronal progenitor cells, and it was shown to promote lineage plasticity in prostate cancer [63]. Acinar PanNECs feature alterations in *CDKN2A* and in the WNT signaling pathway. Whether acinar and ductal PanNECs originate from two different cell types or from the same one and then they follow different trans-differentiating path needs further studies and proper models. Recent findings demonstrate the progression of five different G3 NETs—three PanNETs, one pituitary tumor, and one tumor of unknown origin—towards a NEC phenotype through the acquisition of *TP53* and *RB1* mutations [64]. Notably, in this study, all high-grade NENs retained the same mutations in NET-associated genes such as *DAXX*, *ATRX*, and *MEN1* as the low-grade NETs from which they originated. These data suggest that the acquisition of mutations typical for NECs can drive low-grade tumors toward a more aggressive, high-grade phenotype, and possible shared cell of origin for NETs and NECs. In mice, impairment of the function of tumor suppressor proteins p53 and pRb via β-cell specific expression of the SV40 large T antigen leads to development of malignant insulinomas with NEC features [65]. Importantly, transcriptomic characterization of PanNENs from this RIP1-Tag2 mouse model revealed two different subtypes, the islet tumors (IT) and the metastatic-like primary (MLP) [58, 66]. While the IT comprises insulinomas with low metastatic potential, the MLP are highly invasive, with transcriptomic program similar to liver metastasis. Furthermore, Saghafinia et al. demonstrated that IT tumors can progress into MLP tumors via dedifferentiation process [57].

#### Intestinal NETs

Small intestinal tumors are believed to arise from the enteroendocrine cells of the intestinal tract. A recent multi-omic study on Si-NET revealed 4 different subtypes: epithelial, vesicular, immune and mesenchymal [43]. Comparing transcriptomic profiles of bulk RNA of normal cells of the intestine the authors showed that tumors with epithelial signature display enrichment for enterocyte/goblet cell precursor markers, vesicular tumors were associated with enterochromaffin features, and the mesenchymal subtype was associated with progenitor and stem-like profiles. Interestingly, the mesenchymal type was characterized by the complete loss of pro-glucagon expression, indicating a loss of neuroendocrine differentiation [43]. These findings suggest that Si-NETs can arise from different enteroendocrine differentiation patterns. Rectal NETs are subdivided into L-cell (PP/PYY producing) tumors, which express neuroendocrine markers, and enterochromaffin-cell (serotonin producing) tumors which express markers similar to that of Si-NETs (e.g., chromogranin A) and are thus speculated to have different cells of origin [67].

#### Trans-differentiation in GEP-NENs

In PanNEC it is not unusual to observe expression of exocrine lineage markers such as trypsin, MUC1 and CEA, underlining cell plasticity and possible trans-differentiation [68]. In a mouse model for Pancreatic Ductal Adenocarcinoma, it was shown that neuroendocrine cancer cells trans-differentiation is MYC dependent [69]. Similarly, prostate cancer can evolve into an aggressive subtype acquiring neuroendocrine differentiation often after therapy, also via C-MYC [70]. Rarely, tumors present a mixed cell population including neuroendocrine and non-neuroendocrine cells (MiNENs), where the two components represent at least 30% of the tumor mass [71]. Molecular analysis of MiNENs of the gastrointestinal tract showed that the two components share a core of mutations while also presenting private mutations, suggesting a common cell of origin which diverges during progression in two different entities following additional genetic alterations (reviewed in [72]). Shared mutations usually include *TP53*, *APC*, *KRAS* and *BRAF* and have a higher allele frequency compared to the private ones, suggesting early onset. Interestingly, in general the neuroendocrine component carries a higher allele imbalance, suggesting a more aggressive biology. It has been proposed that often it is the non-neuroendocrine part that trans-differentiates into neuroendocrine, a process which seems to be mediated via c-MYC [73]. However, in a mouse model for Gastric Neuroendocrine Carcinoma, it was shown that MYC deletion in gastric endocrine cells positive for synaptophysin induces development of mixed tumor with both endocrine and exocrine component suggesting trans-differentiation and common cell precursor for both entities [74]. In conclusion, neuroendocrine phenotypes seem to be acquired along progression in tumors with different cells of origin.

## Future directions

### Novel experimental models

Tumor-derived organoids are promising models to study cancer initiation, progression, and treatment. Although elusive for a long period of time because of their slow growth and the difficult culture conditions allowing them to thrive *in vitro*, organoid models for NENs and in particular for NETs have recently been established [23, 31, 75, 76]. Indeed, Kawasaki et al. developed a biobank of 25 patient-derived organoid lines from both, GEP-NETs and GEP-NECs [76]. These organoids reliably replicated the histological and functional characteristics of the original tumors, including their responses to hormone therapies such as somatostatin analogues. Importantly, while 22 organoid lines were successfully established from NECs, only 3 NET lines remained stable long-term, underscoring the persistent challenge of culturing slowly proliferating NETs *in vitro* [76]. Nevertheless, this collective effort marks a significant step toward addressing the scarcity of GEP-NEN models, rooted largely in the rarity of the disease. Organoids of normal tissue can help in dissecting tumor cell of origin. Interestingly, the knock-out of *TP53* and *RB1* in normal colonic organoids does not upregulate the expression of neuroendocrine markers. Further over-expression of the transcription factors ASCL1, NEUROD1, POU3F2, NKX2-5 and TP73, allows neuroendocrine differentiation in this model and acquisition of NEC features [76].

The key ingredient to allow the culture of lung NENs was the addition of protein EGF in the culture medium. However, among lung NETs, this medium allowed only tumors from the A1 group to grow, suggesting that NET cells may be dependent on different growth factors provided by their microenvironment, and thus that on top of the cell of origin, a specific tumor microenvironment of origin is necessary for carcinogenesis. In line with this hypothesis, transcriptomic analyses suggest very different tumor microenvironments for the different lung NET molecular groups [22]. Among the seldom available cell lines, lung NEC are the most abundant [77, 78]. However, the lack of heterogeneity inherent to cell lines impairs their use for investigating biology and treatment.

In summary, recent years have seen the emergence of experimental models that will allow us to study the cell of origin of NENs, but more work is needed to obtain the perfect models. Further *in vitro* and *in vivo* models are needed to clearly elucidate the cell of origin in NENs. Specifically, approaches like forward genetic in different cell types (neuroendocrine and not neuroendocrine) may help to dissect the cell of origin. Tumor micro-environment also may play a crucial role in tumor initiation, hence *in vivo* and *in vitro* models accounting for this aspect are needed. Obtaining realistic models of healthy organs, in particular at different developmental states and recapitulating different parts of the organ (e.g., proximal or distal airway) and rare cell types (e.g., neuroendocrine cells and their progenitor) would be crucial to prove hypotheses about the origin of the different NENs [31].

## Novel technologies

High-resolution sequencing technologies—including single-cell and spatial transcriptomics and proteomics—are revealing normal and tumor tissue heterogeneity and revolutionizing our understanding of organ and cancer development [7, 8]. In particular, single cell resolved spatial sequencing has the potential to reveal the key cellular interactions influencing the transformation from normal to cancer cells [79], potentially elucidating the role of the microenvironment and the modus operandi of potential risk factors such as smoking and air pollution, as well as cell differentiation trajectories [80]. Although common cancers from most organs like the lung (adenocarcinomas) and the pancreas (ductal adenocarcinoma) have already benefitted from the single-cell revolution [81], most NENs, especially NETs, have yet to be sequenced at such a high-resolution. The generation of a pan-cancer NEN atlas will be key in advancing research on the cell of origin of NENs. Another potentially game-changing technology for the investigation of the cell of origin of cancers, including NETs, is deep learning applied to computational pathology [82]. Visual encoders allow to cover patches of whole-slide images (WSI) into simple numerical vectors that capture the essential morphological characteristics of cells from the tissue and the tissue organization itself. Although early supervised models required extensive training on a large series of WSI from a given tumor type, a new generation of self-supervised foundation models trained on hundreds of millions of diagnostic images have emerged in the recent years [83, 84]. These models allow to perform zero-shot learning, without the need for an extensive database of images from the tumor type of interest nor for supercomputers, perfectly fitting the need of rare tumor research such as research on NENs. Importantly, contrary to costly technologies such as single-cell and spatial transcriptomics which studies are restricted to a few samples due to their large cost, once trained, deep-learning models can be easily used on large series of slides. These models have already shown a good accuracy in quantifying cell types within a tissue, identifying rare cell populations, identifying cells with particular genomic alterations [85], and could thus assist in many crucial tasks in the search of the cells of origin of NENs such as helping identify elusive precursor lesions, identifying morphological gradients indicative of cell differentiation trajectories, or identifying pro-tumoral microenvironmental niches.

## Conclusion

In conclusion, the cell of origin of neuroendocrine neoplasms remains to be elucidated. Although promising new hypotheses emerge from recent molecular studies, there is a lack of experimental validation with realistic models such as organoids, and an underuse of current and emerging technological advancements. Elucidation of the cell of origin can provide important insight into driver of tumor formation which can improve treatment. We encourage further studies in this direction to reveal fundamental information that would empower the clinical management of these diseases, informing all stages of prevention, cancer interception strategies, as well as future treatments.

## Declarations

We affirm that both authors have adhered to ethical standards throughout the creation of this manuscript. Ethical approval (not applicable). No ethical approval was required for this review article.
